# Molecular basis of epigenetic regulation in cancer diagnosis and treatment

**DOI:** 10.3389/fgene.2022.885635

**Published:** 2022-08-24

**Authors:** Sonam Tulsyan, Mehreen Aftab, Sandeep Sisodiya, Asiya Khan, Atul Chikara, Pranay Tanwar, Showket Hussain

**Affiliations:** ^1^ Division of Cellular and Molecular Diagnostics (Molecular Biology Group), ICMR- National Institute of Cancer Prevention and Research, Noida, India; ^2^ Symbiosis School of Biological Sciences, Symbiosis International (Deemed University), Pune, India; ^3^ Laboratory Oncology Unit, Dr. B. R. A. Institute Rotary Cancer Hospital, All India Institute of Medical Sciences, New Delhi, India

**Keywords:** epigenetics, cancer, DNA methylation, histone modification, chromatin remodelling, cancer therapies

## Abstract

The global cancer cases and mortality rates are increasing and demand efficient biomarkers for accurate screening, detection, diagnosis, and prognosis. Recent studies have demonstrated that variations in epigenetic mechanisms like aberrant promoter methylation, altered histone modification and mutations in ATP-dependent chromatin remodelling complexes play an important role in the development of carcinogenic events. However, the influence of other epigenetic alterations in various cancers was confirmed with evolving research and the emergence of high throughput technologies. Therefore, alterations in epigenetic marks may have clinical utility as potential biomarkers for early cancer detection and diagnosis. In this review, an outline of the key epigenetic mechanism(s), and their deregulation in cancer etiology have been discussed to decipher the future prospects in cancer therapeutics including precision medicine. Also, this review attempts to highlight the gaps in epigenetic drug development with emphasis on integrative analysis of epigenetic biomarkers to establish minimally non-invasive biomarkers with clinical applications.

## Introduction

Cancer is a multifactorial disease developed as a result of several genetic as well as epigenetic changes. Epigenetics is a process that involves the alteration of gene expression without changing the DNA sequence. It is a Greek word meaning above or over the genome, which was coined by Conard Waddington in 1942 (Waddington, 2012). The process of epigenetics involves structural modifications within the nucleic acids and histones imparting a different chromatin structure and includes three molecular mechanisms like DNA methylation, histone modification, and nucleosome modelling patterns (Ganesan et al., 2019). These epigenetic modifications involve several chemical alterations which are induced by a group of enzymes, called epigenetic tools or players. The enzymes which participate in chemical addition to DNA or histones are known as “writers” whereas “erasers” are those enzymes that are involved in removing chemical tags. All these modifications are interpreted by a separate group of enzymes called ‘readers’ (Biswas and Rao, 2018). Several processes like DNA repair, replication, transcription, translation, post-transcriptional and post-translational regulation, are controlled by epigenetics (Dawson and Kouzarides, 2012). Thus, aberrant expression patterns or epigenomic alterations can lead to misregulation, culminating in cancers (Lu et al., 2020). The interesting part of studying epigenetics is that it is reversible in nature as compared to genetic changes and they only alter how a DNA sequence is read (Jaenisch and Bird, 2003). There are several factors contributing to epigenetic changes in humans like obesity, diet, lifestyle, alcohol, tobacco use, exposure to electromagnetic radiation and environmental pollutants like chromium, cadmium, nickel, benzene, mercury, and arsenic (Jones and Baylin, 2002; Baccarelli and Bollati, 2009; Metere and Graves, 2020).

Research over a decade has focused on promoter DNA methylation and histone modifications as the two main molecular mechanisms that mediate the process of epigenetic regulation in anticancer therapies and biomarker discovery. Studies have demonstrated that the altered DNA methylation genes or patterns can be potentially used as biomarkers for proper cancer screening, diagnosis, and prognosis (Tost, 2009; Karandish and Mallik, 2016; Grayson et al., 2019). Thus, epigenetic biomarker discovery is crucial for early cancer diagnosis, better cancer therapies, precise treatment and effective clinical outcomes. In spite of continuous growth in the discovery and development of biomarkers, advancement in the clinical validation of the approved biomarker is still demanded (Hussain et al., 2022). Many challenges are being faced in the development of a reliable biomarker with clinical applications. One main issue is the incorporation of clinical trial data into routine practice with affordable cost, which is only possible through interdisciplinary collaboration between researchers, clinicians and diagnostics companies.

In addition, the tumour microenvironment (TME) of the cancer cells contains aberrant epigenetic marks which are known to cause a favorable environment for tumor growth (Lodewijk et al., 2021). The existing literature not only emphasizes research on epigenetic regulation and its role in cancer development but also on the interaction of tumour cells with TME. In the context of the above-mentioned facts, this review has shed light on epigenetic changes and the use of integrated network medicine with epigenetics in the development of epimarkers/epidrugs along with several challenges faced during their development. The administration of such anticancer therapies might lead to reverse epigenetics which could be useful in the treatment and management of cancer patients.

## Mechanisms of epigenetic modification

Several molecular mechanisms exist behind epigenetic regulation, including DNA and RNA methylation, histone modifications, and ATP dependent nucleosome remodelling which have been discussed here.

### DNA and RNA methylation

DNA methylation is one of the widely studied epigenetic mechanisms in cancer etiopathogenesis. Aberrant methylation leads to DNA hypermethylation or hypomethylation. In DNA hypermethylation, the process of methylation occurs at the cytosine bases present in the promoter region of genes by a group of enzymes called DNA methyltransferases (DNMTs), including DNMT1, DNMT3a, and DNMT3b (Jones and Baylin, 2002). These enzymes convert cytosine residues to 5-methylcytosine eventually leading to decreased gene expression *via* transcriptional suppression (Fan et al., 2012; Li et al., 2012). On the contrary, DNA hypomethylation indicates overall decrease in the methylation levels as compared to normal cells, and affects the intergenic and intronic regions of the DNA, resulting in chromosomal instability and increased mutation activities (Wilson et al., 2007). The global hypomethylation with hypermethylation of specific gene promoters has already been reported by various studies on cancer (Kurkjian et al., 2008). Thus, inappropriate DNA methylation may lead to altered expression of tumor suppressor genes (deregulation) and/or oncogenes (upregulation) in cancer cells (Kulis and Esteller, 2010). In fact, differences in methylation patterns exist within CpG islands of ∼70% of all mammalian promoters, which have been known to play an important role in transcriptional and post-transcriptional regulation (Robertson, 2005; Tost, 2009). In addition, the introduction of high throughput sequencing has confirmed that 5–10% of abnormally methylated CpG promoter islands are present in various cancer genomes. Also, the hypermethylation of CpG islands in several promoters influences the expression of various noncoding RNAs (ncRNA) as well as messenger RNAs (mRNA), which are known to have a role in cancer progression (Baylin and Jones, 2011). Even, the whole genome sequencing data in several cancers have shown that various somatic mutations exhibit in numerous epigenetic regulators (Forbes et al., 2017).

**FIGURE 1:**
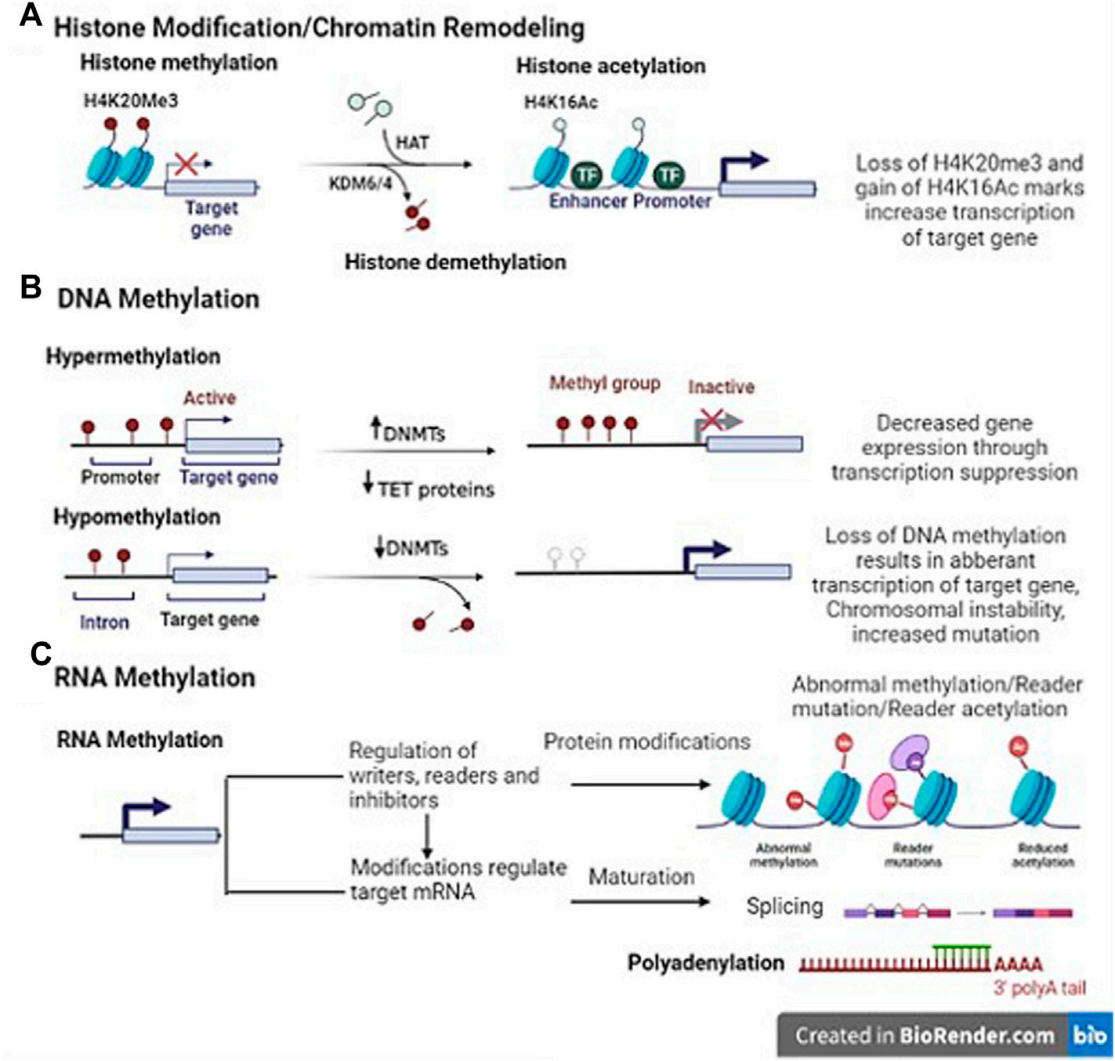
Representative image of epigenetic mechanisms (Template was created by free Biorender.com). **(A)** Histone modification- Histone methyltransferases (HMT) add methyl groups to histones (H4K20Me3). Histone demethylases (HDM)/Lysine demethylase (KDM6/4) remove these methyl groups. It is associated with both gene expression and silencing. Histone acetylation; The addition of an acetyl group on H3K9Ac (lysine 9 histone H3) in enhancer/promoter region by histone acetylase (HAT) enzyme. Histone deacetylase (HDAC) interact with transcriptional repressor (TR) to remove the modifications. **(B)** DNA methylation- DNA methyltransferases (DNMTs) add methyl group in the promoter region of genes. On the contrary, DNA hypomethylation indicates overall decrease in the methylation levels as compared to normal cells, and affects the intergenic and intronic regions of the DNA, resulting in chromosomal instability and increased mutation events **(C)** RNA methylation- Indirect translational repression by miRNA causes deadenylation, in which the 3′ poly(A) tail of an mRNA is removed, leading to increased mRNA degradation. The miRNA–mRNA interaction can lead to several modes of direct translational repression.

A less studied epigenetic process is RNA methylation. It is about seven times greater than DNA methylation. These modifications result in mRNA localization and transcript degradation (Zaccara et al., 2019). With the advent of next generation sequencing and the discovery of RNA methylation-related proteins, it is easy to comprehend that methyl modifications at mRNA level may affect the cellular processes resulting in human diseases. Presently, over 150 different RNA modifications have been observed, of which the N^6^-methyladenosine (m^6^A) modification is the most abundant and it is recognized by RNA binding proteins that affect many characteristics of mRNA function (Schwartz et al., 2014; Linder et al., 2015). Similar to modifications at the DNA methylation level, alterations at RNA level affect the epigenetic regulation of gene expression (Figure 1).

### Histone modification

We are already familiar with the chromatin structure which involves wrapping of DNA on histone octamer -2 subunits each of H2A, H2B, H3 and H4 proteins joined together by H1 proteins. Histone modification takes place at the amino-terminal tail of these histones *via* the process of acetylation, methylation, phosphorylation, ADP-ribosylation, or ubiquitination. Several enzymes are known to catalyse the above-mentioned processes. The addition of acetyl, methyl, phosphate group etc. to histone amino-terminal tail is performed by histone acetyltransferases (HATs), histone methyltransferases (HMTs), and histone kinases. These epigenetic marks are known as writers and act as transcriptional co-activators. On the contrary, erasers [histone deacetylases (HDACs), histone demethylases (HDMs), phosphatases] function as transcriptional co-repressors by removing these groups from histone end (Li et al., 2020). All these enzymes are involved in the simultaneous opening and closing of chromatin structure, which is necessary for gene expression to occur (Figure 1). Aberrations in these enzymes may lead to altered gene transcription and post-transcriptional modifications, thereby resulting in cancer.

### ATP dependent chromatin remodelling

The DNA nucleosome interactions can be modified (histone ejection, removal and incorporation) through chromatin remodelling complexes by ATP hydrolysis. These chromatin-remodelling complexes can be classified into switching defective/sucrose nonfermenting (SWI/SNF), chromodomain-helicase DNA-binding protein (CHD), imitation SWI (ISWI), and INOsitol requiring mutant 80 (INO80) complexes. The catalytic subunit of these complexes performs DNA translocation along with the histone core of the nucleosome (Clapier et al., 2017).

The SWI/SNF complexes are one of the most widely studied ATP dependent chromatin remodelling complexes. They are found to be mutated in 25% of human cancers (Mittal and Roberts, 2020) and are playing an essential role in chromatin remodelling by positioning nucleosomes. Their catalytic activity is known to be associated with SMARCA4/2 proteins. Numerous studies also suggest that SWI/SNF complexes are involved in the regulation of cell progression, cell motility, and nuclear hormone signalling (Wilson and Roberts, 2011). The SWI/SNF complex was found to be altered in 33–42% of pancreatic cancer cases by whole-exome sequencing studies (Shain et al., 2012; Witkiewicz et al., 2015).

The next important complex is ISWI which mobilizes nucleosomes by helping the transcription factors to bind a nucleosome-free DNA. Unlike SWI/SNF complex, ISWI is held to nucleosomes by a SANT and a SLIDE domain (Grüne et al., 2003). As reported in the literature, ISWI complexes have a key role in DNA repair and recombination (Aydin et al., 2014). In humans, two ISWI subunits namely sucrose nonfermenting 2L (SNF2L) and sucrose nonfermenting 2H (SNF2H) ATPases are identified. It has been noticed that SNF2H suppressed the oncogene ras in human cells (Andersen et al., 2006). A tissue microarray study on 78 paraffin wax-embedded prostatic tissues observed a significant increase in ISWI (SNF2L and SNF2H) proteins in prostatic intraepithelial neoplasia and prostate adenocarcinoma (Mohamed et al., 2007). To date, no clinical trials have been performed to unravel the potential of these small molecules as epigenetic biomarkers in cancer therapies.

## Epigenetic diagnostic biomarkers

**FIGURE 2:**
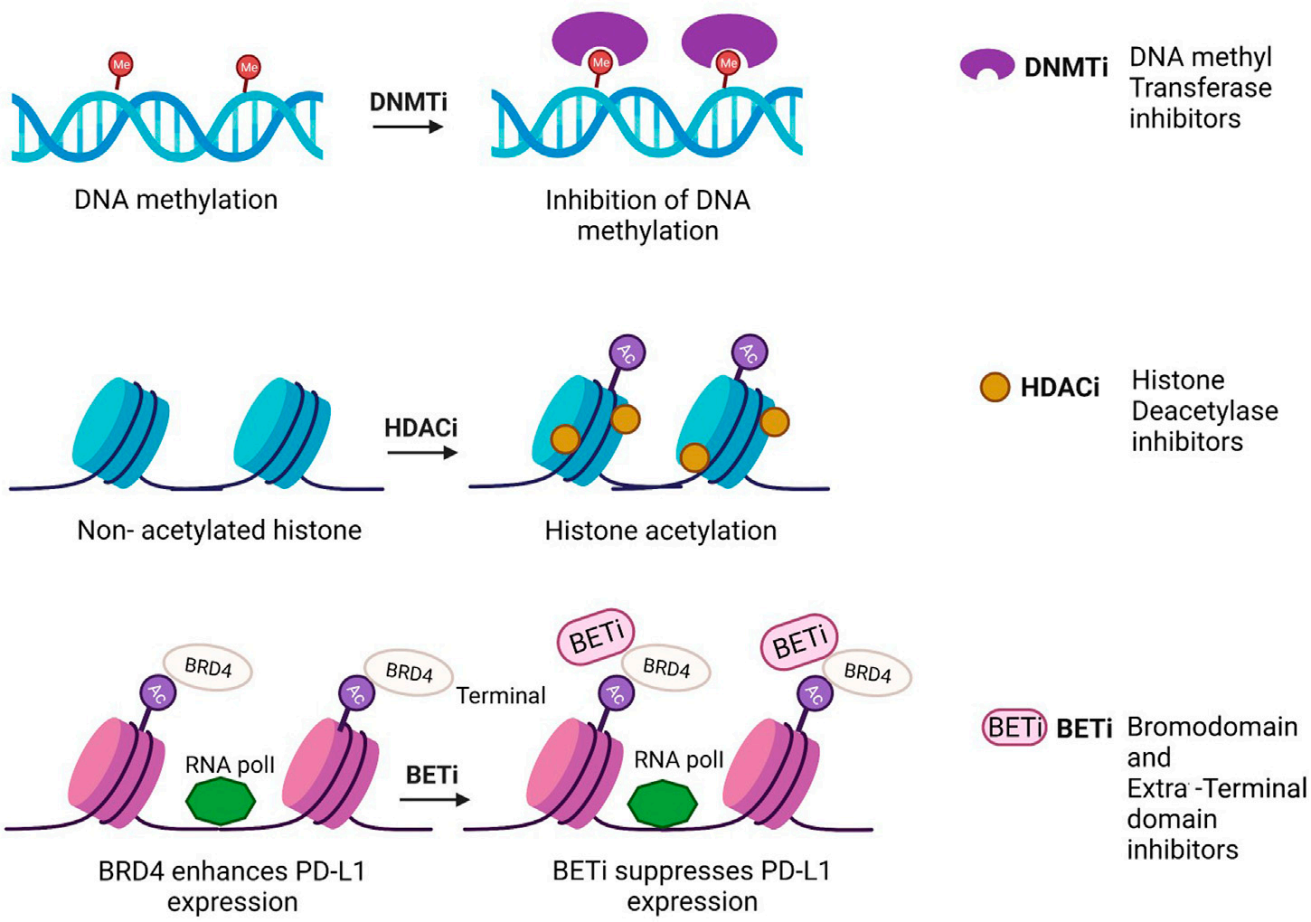
Mechanism of action of epidrugs in anticancer therapies [Icons were created by Biorender.com (accessed on February 9th 2022)].

**FIGURE 3:**
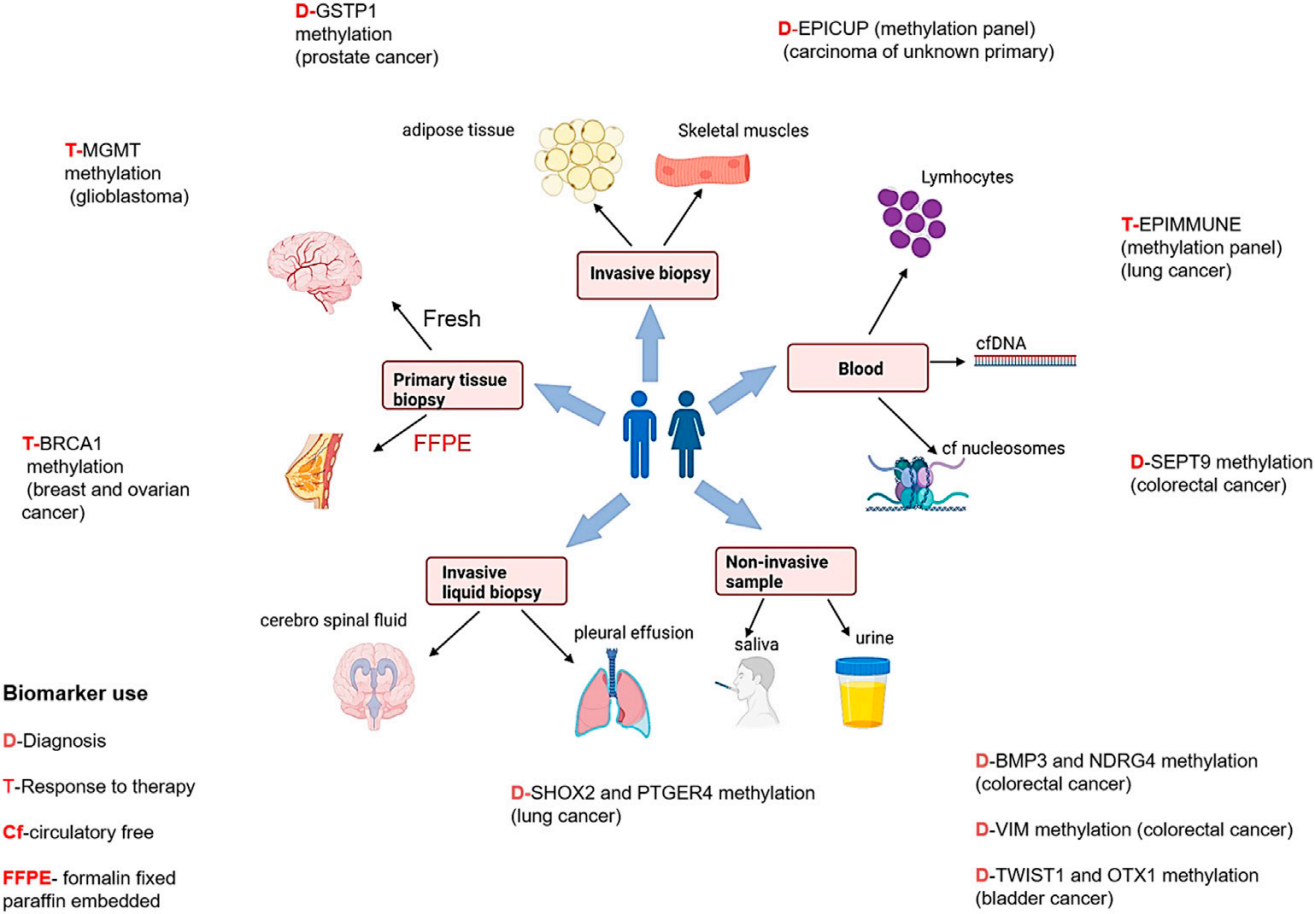
Epigenetic biomarkers and different sample types for diagnosis, prognosis, and treatment therapies in different cancers [Icons were created by Biorender.com (accessed on January 28th 2022)].

Epigenetic changes like DNA methylation and histone modification detected in early tumorigenesis and cancer progression have been proposed as biomarkers for early cancer detection, tumor prognosis, and treatment response (Figure 2). They are rarely translated into biomarkers for clinical practice, even though there have been major advances in the characterization of cancer. Due to stability in body fluids like urine and serum, which have a great opportunity for assay development to assistance in patient’s treatment, the epigenetic changes act as innovative cancer biomarkers. Recent studies have identified various epigenetic cancer biomarkers that have already been commercialized. However, further validation studies are required to take it to the clinics. Over here, epigenetic diagnostic and prognostic biomarkers that are most promising for the most common cancers have been discussed (Figure 3).

### Prostate cancer

Prostate cancer is the second most common cancer (14.8%) in men and the fourth leading cause of cancer related death (6.6%) (Sung et al., 2021), globally. Prostate cancer is quite heterogenous and lethal disease at clinical level, which makes it impervious not only to diagnose it early but also for evaluating the threat that a given prostate cancer bears to its host (Jain et al., 2014). Therefore, further studies are required to develop epigenetic biomarkers to meet these goals. In 95% of prostate cancer patients, higher expression of PCA3 and ncRNA have extensively been reported in blood samples. Commercially available PROGENSA™, prostate cancer biomarker (Durand et al., 2011) quantifies PCA3 expression ratio normalized as input control for prostate specific antigen (PSA) mRNA. For early detection of prostate cancer by semi non-invasive method, PCA3 testing may be useful, thus also avoiding unnecessary prostate biopsy. The most promising prostate cancer epigenetic biomarkers are DNA methylation and glutathione S-transferase pi gene (GSTP1) promoter hypermethylation (Maldonado et al., 2014).

### Glioblastoma

Few studies have reported a correlation of promoter methylation at the methylguanine-DNA methyltransferase (MGMT) gene with favorable treatment outcomes in glioblastoma patients treated with temozolamide, suggesting its possibility to be used as an epigenetic biomarker (Donson et al., 2007; Rosas-Alonso et al., 2021; Kim et al., 2022).

### Colorectal cancer

Studies have shown that high levels of hypermethylated DNA exists in colorectal cancer which leads to genomic instability (Toyota et al., 1999). The methylation status of five genes- CACNA1G, IGF2, NEUROG1, RUNX3, and SOCS126 may identify CpG island methylator phenotype (CIMP) positive colon cancers, which are characterized by high incidence of p16 and THBS1 methylation and frequent KRAS and BRAF mutations (Lao and Grady, 2011; Zhang et al., 2021). One of our published studies observed that RASSF1A, FHIT and MGMT gene methylation patterns may be used as markers in diagnosing colorectal cancer (Sinha et al., 2013).

### Esophageal cancer

A recent epigenomic study has shown differential methylation patterns in several genes which may account for esophageal cancer development and in future can be realised as diagnostic biomarkers (Lin et al., 2018). An Indian study observed promoter methylation in 52% of histopathologically confirmed tumor tissues and the methylation frequency increased with higher histological grades of the cancer (*p* = 0.0001) (Salam et al., 2009).

### Bladder cancer

In 2021 (Sung et al., 2021), the incidence rate of bladder cancer was 3.3%, and the mortality rate of 2.0% globally. At present no accurate diagnostic or prognostic biomarkers are commercially available. Some biomarkers representing higher sensitivity than cytology (Letelier et al., 2012) have been reported based on methylation. As per some studies, based on genome-wide characterization, bladder cancer cells demonstrated that VIM, GDF15, and TMEFF2 show 94% sensitivity and 100% specificity in urine samples (Costa et al., 2010). The data of other epigenetic alterations like histone modification in bladder cancer is infrequent.

### Breast Cancer

Several studies have suggested alterations in histone-modifying enzymes like enhancer of zeste homolog 2(EZH2). This enzyme is encoded by EZH2 gene, which participates in histone-methylation and transcriptional repression. EZH2 has significantly reduced expression of histone in breast cancer (Yomtoubian et al., 2020). Another one is Protein Arginine Methyltransferase (PRMT1). It is also a histone modifying enzyme which plays an important role in the epithelial-to-mesenchymal transformation (EMT) of breast cancer cells (Mathioudaki et al., 2011) in the development of cetuximab sensitivity in triple-negative breast cancer (TNBC) cell lines (Gurdal et al., 2019) with high grade malignancy and poor prognosis (Bianchini et al., 2016). The role of SETD7 (SET Domain Containing 7), lysine methyl transferase in post translational modification of non-histone protein and having prognostic as well as negative effects with tumorigenesis and poor prognosis in patients (Huang et al., 2017) with the expression of lysine methyltransferase SETD7 has been suggested. It is a potential promoter of the antioxidant pathway balancing the cytotoxic effect of oxidative stress. Further validation, for histone modification-based biomarkers is still required.

Stirazaker et al., 2015 divided TNBCs into different epigenetics groups viz. high, intermediate, and low-risk groups based on epigenetic subtypes and the methylated region that is correlated with the progression of the disease. One of the recent studies has also confirmed a correlation between shorter periods of reduction in analysed TNBC-samples (Stirzaker et al., 2015) and hypermethylation of gene regions. Hypermethylation provides explanations and evidence for clinical threat and helps in treatment planning in patients having a higher risk of recurrence.

### Ovarian cancer

The worldwide incidence and mortality rates of ovarian cancer in the year 2021 were 1.1% and 2.3%, respectively (Sung et al., 2021). Histone modifications by acetylation with aberrant tubulin protein expression, reduction of PACE3 expression, silencing of survivin (BIRC5), upregulation of pRb tumor suppressor gene and CDKN1 (Cyclin-dependent Kinase) were reported in ovarian tumor formation. Furthermore, the overexpression of Histone Deacetylase Enzymes HDAC3, and loss of H3K27me3 (an epigenetic modification of DNA histone protein H3) was reported to be associated with prognosis (Li et al., 2021) and higher stages of tumor in ovarian cancer. H3K4me3 plays an important role in the transcriptional repression of tumor necrosis factor TNFRSF11B and upregulate the H3K27me3 (Chapman-Rothe et al., 2013). The loss of RNF20 (Ring Finger Protein 20) and H2Bub1 (H2B monoubiquitination) to the progression of ovarian tumors by chromatin remodelling has been reported by a very recent study (Hooda et al., 2019). Tang et al. (2018) revealed that AMPK (Activated Protein Kinase) moderate the repression of H3K27me3 after treatment with metformin and expressed its usefulness in the treatment of ovarian cancer cases. A study also reported that EZH2 facilitates TIMP2 (Tissue Inhibitor of Metalloproteinase 2) and ADP-Ribosylarginine hydrolase 1 (ARH1) by DNA methylation and H3K27me3, which leads to ovarian cancer metastasis. Their inhibitors could thus be used as potential epigenetic biomarkers for the early detection and diagnosis of cancer after proper clinical studies and validation of the same.

Research has recognized the role of epigenetics in developing drug resistance thereby affecting cancer treatment. Cacan et al. (2016) reported that the loss of apoptosis antigen 1 expression impacts drug resistance, which is mediated by histone deacetylase 1 (HDAC1) in chemoresistant ovarian cancer cells (Cacan, 2016). Using ChIP-sequencing, Curry et al. (2018) identified H3K27me3 and H3K4me3 methyltransferases in the promoter region in tumor cases which were acquired resistance for pre and post platinum and showed that these genes are involved in epigenetic silencing during chemotherapies and are prone to hypermethylation thus providing novel awareness to prevent disclosure of drug resistance (Curry et al., 2018).

A recent study has described hypomethylation of developmental genes MSX1, DAXX and TMEM88. mRNA expression of these developmental genes is associated with platinum resistance and inversely correlated with promoter methylation in ovarian cancer patients by treatment with Guadecitabine (DNA methyl transferase inhibitor) and cisplatin (Bonito et al., 2016; de Leon et al., 2016).

DNA methylation regulates epithelial mesenchymal transition (EMT) by lncRNA (HOTAIR) and it is a sign of resistance to carboplatin (Singh et al., 2019). A study has also described how DNA methylation targeted genome scale strategies could prevent the formation of tumors, for example Guadecitabine facilitated inducing hypomethylation, activates tumor suppressor genes and affects metabolic and immune responses to contributing platinum drug desensitization in ovarian cancer. It may help in improving of patients survival outcomes with ovarian cancer (Fang et al., 2018). An epigenetic study described that the methylation of Zinc Finger protein 671 (ZNF671) can serve as a prognosticator for the early relapse of ovarian tumorigenesis and correlates with disease aggressiveness and progression (Zhang et al., 2019). Epigenetic inhibitors used for combinational therapies, would possibly be most effective by repair of pathways associated with drug response for chemo-desensitization of resistant tumors and would consequently implicate improved survival outcomes as well as personalized treatment for various cancers**.**

## Epigenome-targeted therapies

**FIGURE 4:**
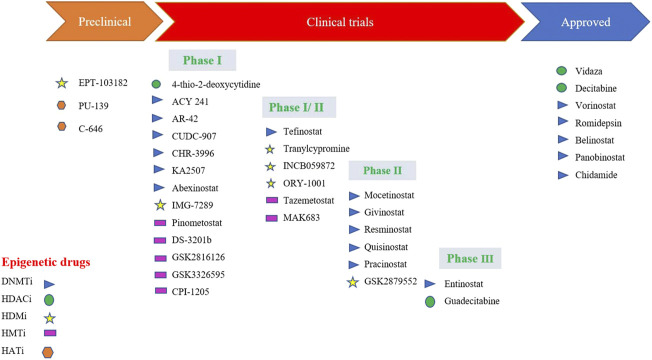
Epidrugs in preclinical and clinical trials for cancer therapy [Icons were created by Biorender.com]. (DNMTi - DNA methyl Transferase; HDACi—Histone deacetylase inhibitor; - HMTi -Histone methyltransferase inhibitors; HDMi- Histone demethylase inhibitors and HATi -Histone acetyltransferase inhibitors).

Quite a few epidrugs are approved for the treatment of several cancers. These epidrugs are the inhibitors of DNA methyltransferase (DNMTi) and histone deacetylase (HDACi) enzymes (Figure 4). The first US-FDA approved epigenetic drug is 5- azacitidine (Azacitidine), a DNMTi which is used in the treatment of myelodysplastic syndromes (MDS) and acute myeloid leukaemia (AML). Even combination therapies including both DNMTi and HDACi are widely inspected in the treatment of MDS, AML and chronic myelomonocytic leukaemia (CMML) (Blagitko-Dorfs et al., 2019). However, clinical results for such a combination of inhibitors are controversial (Thurn et al., 2011). The major reason being the lack of large sized cohort studies. Now, the research on epidrug development has expanded its boundary to targeted therapy, shifting the focus on the presence of activating mutations in epigenetic players, especially histone methyltransferases. It has been found that the evolutionarily conserved histone modifier EZH2 is mutated in several cancers. Another inhibitor of EZH2, Tazemetostat (TAZVERIK, Epizyme, Inc.) was approved by US- FDA in June 2020 for treating adult patients with relapsed or follicular lymphoma with EZH2 positive mutations.

One of the main problems in the application of epidrugs is that the drug binds to other targets rather than its own target. This is called “off-target effects” in epigenetic therapy. Growing epi-research has shown that the use of synthetic lethal approaches might result in apoptosis. In this approach, two inactive genes which synergistically result in apoptosis are selected and combined. These epidrugs are delivered to target synthetic lethal partners having genetic mutations in cancer cells. However, these drugs are less/not toxic to non-cancerous cells with no mutations, resulting in more precise therapy. One such epidrug is the inhibitor of the histone methyltransferase DOT1L (disrupter of telomere silencing 1-like), Pinometostat which specifically kills the MLL-fusion leukaemia cells (Marcos-Villar and Nieto, 2019). Another epidrug used in the treatment of lung cancer with a specific DNA hypomethylation is GSK2879552. It is an inhibitor of lysine-specific histone demethylase 1A (LSD1) (Smitheman et al., 2019). GSK2879552 treatment results in the increase of H3K4 methylation, thereby reducing tumour potential (Fang et al., 2019).

Interestingly, DNA methylation biomarker technology is being employed in circulating free DNA present in body fluids to detect cancers. In the year 2017, “Epi proLung^®^” assay has received the *Conformité Européenne* (CE) mark as *In Vitro* Diagnostic (IVD) test for lung cancer diagnosis. It is based on methylation analysis of SHOX2 (Short Stature Homeobox 2) and PTGER4 (the prostaglandin E receptor 4) genes (Beltrán-García et al., 2019). Numerous reports have shown increased promoter methylation of *SEPT9, Vimentin*, and *NDRG4* gene in colorectal cancer. The US- Food and Drug Administration (FDA) has also approved non-invasive DNA methylation tests of these genes for early colorectal cancer screening programmes (Ned et al., 2011; Lamb and Dhillon, 2017).

Another CE-IVD marked test, miRpredX-31-3p kit (IntegraGen S.A., France) is based on the quantification of miR-31-3p expression levels. It is used to recognise metastatic colorectal cancer patients who can benefit from anti-EGFR (epidermal growth factor receptor) therapy (Ramon et al., 2018). Thus, an effective evolution of epidrugs in cancer therapeutics can be seen from inhibitors to combination therapies to non-invasive diagnostic assays. However, the area of epigenetics still needs to be explored in precision oncology for effective cancer treatment and management.

## Epigenetics and integrated network medicine

The future of epidrug development involves the use of integrated network medicine with epigenetics, where several analytical methods like protein-protein interaction (PPI) networks, correlation-based networks and gene regulatory networks are utilized to roll out key genes, relevant regulatory and co-regulatory networks in causing disease pathogenesis (Silverman et al., 2020; Sarno et al., 2021). A group at Stanford University, United States has developed the Genomic Regions Enrichment of Annotations Tool (GREAT) for functional enrichment analysis of DNA binding events across the entire genome, which is useful in identifying gene-regulatory networks and subnetworks in epigenomics data analysis (McLean et al., 2010). Another integrative epigenome-transcriptome-interactome tool called Functional Epigenetic Modules (FEM), identified HAND2 methylation as an important epigenetic alteration in the development of endometrium cancer (Jones et al., 2013; Jiao et al., 2014). In addition, integrative analysis on epigenetic modifications and their effect on gene expression can be performed using Epigenetic Module based on Differential Networks (EMDN) algorithm (Ma et al., 2017). These frameworks could be utilized directly from epigenomic data to unravel co-regulatory networks responsible for causing the disease.

Zheng et al. (2020), used deep neural network (DNN) algorithm to predict cancer diagnosis in the DNA methylation data of 7,339 patients of 18 different cancer origins from The Cancer Genome Atlas (TCGA) (Zheng and Xu, 2020). Recently, weighted correlation network analysis (WGCNA) of 201 patients in a TCGA prostate cancer dataset revealed hypermethylation of FOXD1 might promote poor prognosis (Zhang et al., 2020). Another study yielded 13 genes epigenetic signature that stratified breast cancer patients into low and high-risk groups by using WGCNA analysis and single sample gene set enrichment analysis (ssGSEA) (Bao et al., 2019). One more tool named SWItchMiner (SWIM) is being used to identify potential therapeutic targets when applied to large panel of cancer datasets from TCGA (Paci et al., 2017).

**FIGURE 5:**
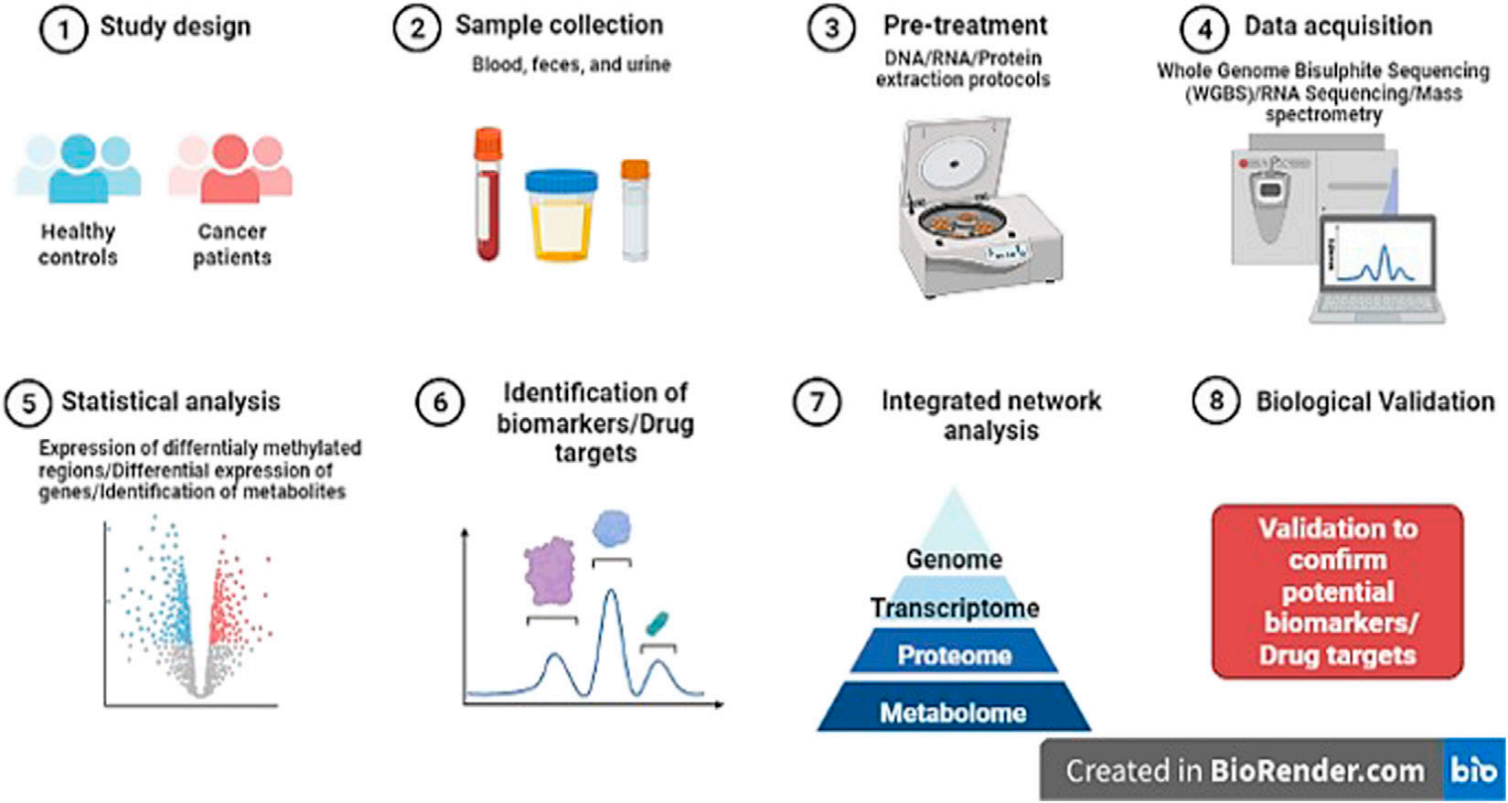
Diagrammatic representation of research pipeline on the discovery of novel potential epi-markers in human cancers (Template was created by free Biorender.com).

Thus, network medicine might advance the field of epigenomics as it is possible to rule out the co-regulatory networks of DNA methylation (Figure 5). However, its clinical application is still lacking accompanied by the challenges of integration of epigenomics data in multi-omics.

## Challenges and way forward for epidrug development

Epidrug development is accompanied by its own set of challenges that needs to be addressed for the establishment of locus-specific, highly sensitive and cost-effective biomarkers. The main obstacle is to comprehend the “casualty” of epigenetics, meaning that whether the epigenetic abnormality is a result of malignancy or malignancy itself is caused due to these variations. It is crucial to decipher the link between epigenetic differences and cancer progression to establish a biomarker with potential utility in the cancer clinics. This is only possible by pursuing special cohort studies where epigenome profiling can be maintained before the start and after the end of the disease. The high cost of such studies limits their application.

Another challenge is the lack of locus specificity which might cause epigenome-wide “off-target” effects leading to the loss of important gene function and can be resolved using new epigenome editing approaches (Du et al., 2015). With the introduction of proteolysis targeting chimera (PROTAC) drug design approach, epidrugs specific to genetically altered chromatin players can be developed, thereby offering precise cancer therapies approach in treatment (Majchrzak-Celinska et al., 2021).

It is understandable that the epigenetic changes, especially the methylation patterns are very informative in establishing both diagnostic and prognostic biomarkers. But the problem lies in the complex assay systems, imprecise reproducibility, inadequate clinical validation, and false discovery of these biomarkers (Lorincz, 2011). Therefore, implementing clinical epigenetics for the benefit of public health is the main goal of epigenetics research. However, it is restricted due to the variable cellular composition of epigenomic profiles of bulk cell populations. To resolve this issue, single-cell methods should be undertaken to provide resolution of DNA at single-cell level like 5-methylcytosine or 5-hydroxymethylcytosine. Also, computational algorithms can be used to correct variable cellular composition by comparisons to reference epigenomes. Another biological challenge is the limited knowledge of complex epigenome with small sized studies. In addition to the epigenome, epitranscriptome should be studied to rule out potentially modified RNA molecules in cancer, whose potential as epigenetic marks can be exploited before clinical application (Salam et al., 2009). As epigenetic modifications are dynamic, it is vital to consider all epigenetics layers using multiomics approaches along with integrated network medicine for epidrug development.

## Conclusion

It is well established that there is a link between cancer and epigenetics. Some epigenetic drugs have already been approved by US-FDA and many more epidrugs are under development for appropriate cancer detection and treatment. Furthermore, there is a scope for epigenetics-based cancer therapies delineating the tumor heterogeneity in different cancers with precision, that should focus on cell-cell behaviour in TME. In addition, epigenetic research should focus on network and precision medicine approaches for the discovery of novel biomarkers so that they can be safely translated to the clinic after proper clinical trials. Thus, proper identification of the epigenetic landscape behind the cancer progression and establishing therapeutic drugs is the future of epigenetics in cancer without forgetting to overcome the challenges faced in effective epidrug development.
